# Construction and Validation of a Clinical Pregnancy Outcome Prediction Model for Infertility Treatment Using IVF/ICSI: A Retrospective Study Based on 11,449 Cases

**DOI:** 10.3389/fendo.2026.1594250

**Published:** 2026-01-28

**Authors:** Yan Guo, Yonghan Luo, Yunxiu Li, Yun Feng, Jie Zhang, Jiacong Yan, Ying Ai, Jiahong Tan, Han Zhao, Xiu Zou, Man Li, Ze Wu, Lifeng Xiang, Xueshan Xia

**Affiliations:** 1 Department of Reproductive Gynecology, NHC Key Laboratory of Healthy Birth and Birth Defect Prevention in Western China, First People’s Hospital of Yunnan Province, Kunming, Yunnan, China; 2 Department of Reproductive Gynecology, The Affiliated Hospital of Kunming University of Science and Technology, Kunming, Yunnan, China; 3 Faculty of Life Science and Technology, Kunming University of Science and Technology, Kunming, Yunnan, China; 4 Second Department of Infectious Disease, Children's Hospital Affiliated to Kunming Medical University (Kunming Children's Hospital), Kunming, Yunnan, China; 5 Yunnan Key Specialty of Pediatric Infection (Training and Education Program)/Kunming Key Specialty of Pediatric Infection, Kunming, Yunnan, China; 6 Department of Reproductive Medicine, NHC Key Laboratory of Healthy Birth and Birth Defect Prevention in Western China, First People’s Hospital of Yunnan Province, Kunming, Yunnan, China; 7 School of Public Health, Kunming Medical University, Kunming, Yunnan, China

**Keywords:** assisted reproductive technolog, clinical pregnancy, infertility, IVF/ICSI, prediction model

## Abstract

**Background:**

Infertility is a prevalent global reproductive health issue. *In vitro* fertilization (IVF) and intracytoplasmic sperm injection (ICSI), as pivotal assisted reproductive technologies, are widely implemented in clinical practice. However, clinical pregnancy outcomes following IVF/ICSI are influenced by various factors, making accurate prediction essential for optimizing treatment strategies.

**Objective:**

To develop and validate a predictive model for clinical pregnancy outcomes following IVF/ICSI treatment.

**Methods:**

A retrospective analysis was conducted on clinical data from 154,307 patients who underwent assisted reproductive treatment due to infertility at the First People’s Hospital of Yunnan Province. Based on inclusion and exclusion criteria, 11,449 patients who underwent IVF/ICSI were included. Key predictors were identified using LASSO regression. A Nomogram scoring system was developed for an intuitive visualization of individualized prediction results. Model performance was evaluated using the area under the receiver operating characteristic (ROC) curve, calibration curves, decision curve analysis (DCA), and clinical impact curves.

**Results:**

LASSO regression identified eight critical predictors influencing clinical pregnancy outcomes: male age, antral follicle count (AFC), Day 3 follicle-stimulating hormone (FSH) level, endometrial thickness, female age, number of usable embryos, number of high-quality blastocysts, and number of embryos transferred. The predictive model demonstrated excellent performance in both the training and validation cohorts, with AUC values of 0.839 [95% CI (0.825, 0.852)] and 0.827 [95% CI (0.817, 0.835)], respectively, indicating strong discriminatory ability. Calibration curves confirmed a high degree of consistency between predicted probabilities and actual outcomes. Decision curve analysis revealed substantial net clinical benefit across various risk thresholds, while clinical impact curves further validated the model’s practical applicability in clinical settings.

**Conclusion:**

This study identified key factors influencing clinical pregnancy outcomes following IVF/ICSI treatment, including male age, antral follicle count (AFC), Day 3 follicle-stimulating hormone (FSH) level, endometrial thickness, female age, number of usable embryos, number of high-quality blastocysts, and number of embryos transferred. This model serves as a scientifically sound decision-support tool for clinicians in the management of infertility treatment with IVF/ICSI.

## Introduction

Infertility is defined by the World Health Organization (WHO) as the inability to achieve a successful pregnancy after at least 12 months of regular, unprotected intercourse. It has become an increasingly severe global health issue, affecting approximately one in six couples of reproductive age and imposing significant psychological, social, and economic burdens on individuals, families, and society at large (1). Assisted Reproductive Technology (ART) has emerged as a critical approach to treating infertility, bringing new hope to infertility patients and creating the possibility of becoming parents. To date, over 10 million children have been born worldwide through ART (2). Among them, intracytoplasmic sperm injection (ICSI) is a complementary and improved method to *in vitro* fertilization (IVF). Both of them have become the most commonly used and effective options due to their mature technical systems and wide range of indications (3, 4). However, despite continuous technological advancements, the success rate of IVF/ICSI treatment remains limited, with an implantation rate of about 60% (5) and a live birth rate (LBR) of only 25-30% (6). There are significant differences in clinical pregnancy outcomes among different patients (7–9). Therefore, accurately predicting the pregnancy success rate of IVF/ICSI treatment has become a critical challenge in clinical practice.

Most existing studies on pregnancy outcomes are based on retrospective research to explore their risk factors (10–12), but such studies often have small sample sizes, and the clinical practicality of predicting pregnancy outcomes based on a single clinical indicator is poor. AI-based machine learning methods can integrate complex and high-dimensional data and are widely cited in the prediction of clinical diseases (13–15). By establishing a simple and visual predictive model through machine learning, doctors can predict individual disease risk based on clinical data such as a single patient’s medical history and laboratory tests, thereby formulating personalized treatment plans. Currently, clinical predictive models for pregnancy outcomes typically consider multiple factors, including female age, duration of infertility, body mass index (BMI), ovarian reserve (such as antral follicle count and anti-Müllerian hormone levels), previous pregnancy history, and sperm parameters (16, 17). For example, Shingshetty et al. pointed out that female age is the most important factor affecting IVF success rates, and almost all relevant studies have included it in their models (17). In addition, BMI and ovarian reserve are also considered important predictive factors (18, 19). These factors have been repeatedly validated in different studies and have been shown to significantly impact the success rates of IVF/ICSI. However, these studies generally suffer from small sample sizes and incomplete variable selection, leading to limitations in the stability and clinical applicability of predictive models. Therefore, there is an urgent need to construct more accurate and generalizable prediction models based on large sample data.

Based on the above reasons, this study aims to develop an efficient and accurate IVF/ICSI pregnancy outcome prediction model using a large sample retrospective data of 154,307 cases. Compared to previous studies, our research goal is to provide clinical doctors with a scientific decision-making basis and help patients formulate individualized treatment plans. Through this study, we hope to provide strong support for the clinical practice of assisted reproductive technology, ultimately promoting the realization of personalized diagnosis and treatment, improving patient pregnancy outcomes, and optimizing the use of medical resources.

## Materials and methods

### Study population

**Figure 1:**
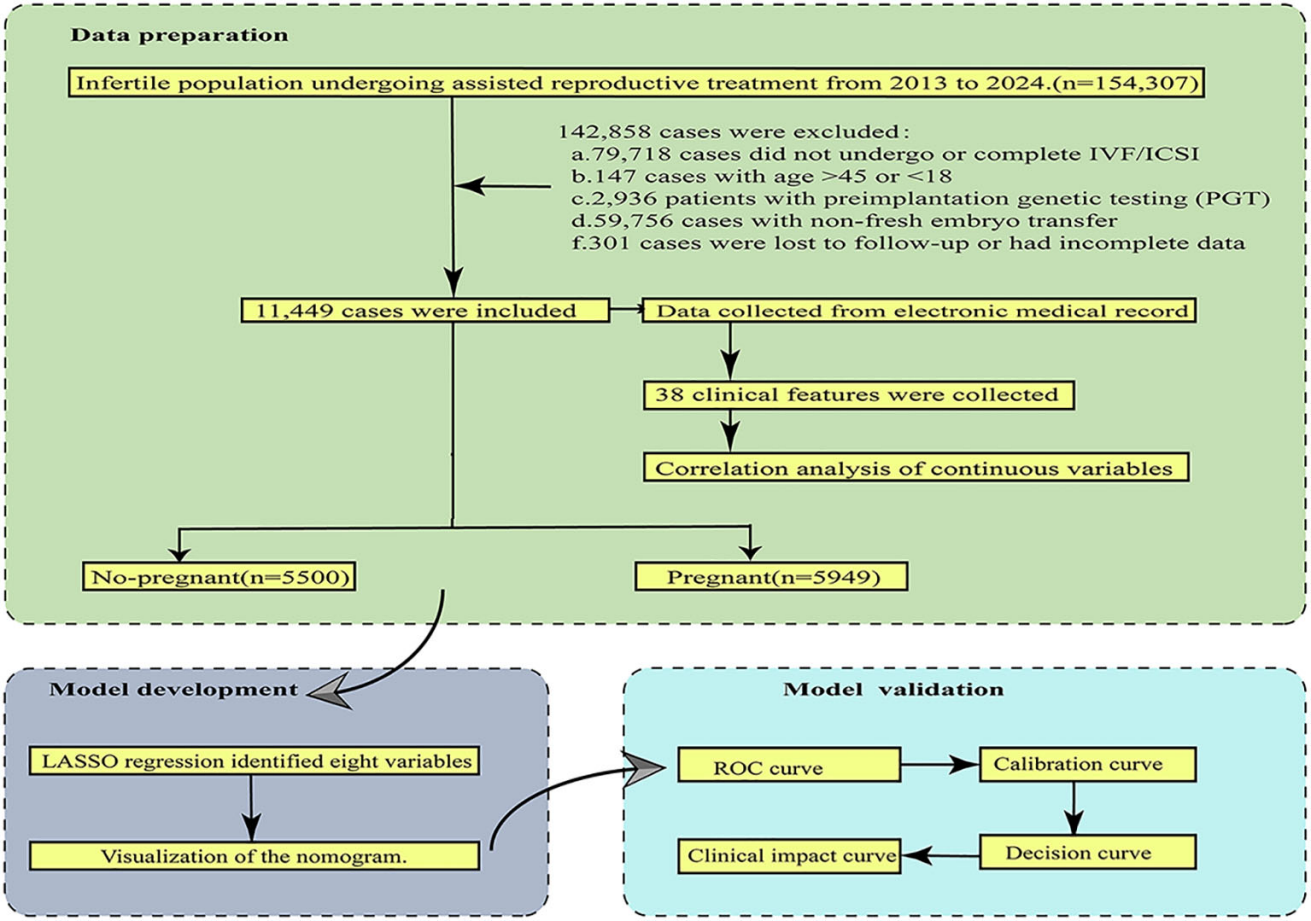
A flowchart illustrating the model development and validation process for predicting clinical pregnancy outcomes in infertility treatment using IVF/ICSI.

Clinical data were collected from patients undergoing ART for infertility at the Reproductive Medicine Center of the First People’s Hospital of Yunnan Province between January 2013 and January 2024. The study flow chart is illustrated in **Figure 1**. The study protocol was approved by the Ethics Committee of the First People’s Hospital of Yunnan Province (Ethics Approval No.KHLL2023-KY120).

### Inclusion and exclusion criteria

#### Inclusion criteria

1. Patients meeting the international and domestic definitions of infertility (1).
2. Patients undergoing ART.

#### Exclusion criteria

1. Patients who did not undergo IVF/ICSI treatment or did not complete the treatment process.
2. Patients aged >45 years or <18 years.
3. Patients undergoing preimplantation genetic testing (PGT).
4. Patients receiving non-fresh embryo transfers.
5. Patients lost to follow-up or with incomplete data.

Based on the outcomes following IVF/ICSI treatment, specifically “clinical pregnancy,” patients were categorized into the Pregnancy Group(n=5949) and the Non-Pregnancy Group (n=5500).

### Data extraction

The study retrospectively collected wide range of clinical and laboratory parameters. General patient information included male age, male BMI, female age, and female BMI. Infertility-related data comprised the causes of infertility (male factor, female factor, couple factor, unexplained), types of infertility (primary and secondary), and the duration of infertility. Female baseline clinical parameters included endometrial thickness (EMT), Day3 follicle-stimulating hormone (FSH) level, antral follicle count (AFC), and the number of mature metaphase II (MII) oocytes. Male semen quality and functional parameters were recorded, including semen volume, sperm concentration, total motility, viability, abnormal morphology, acrosin activity, and DNA fragmentation index (DFI). Fertilization methods were classified IVF and ICSI. Oocyte retrieval and fertilization parameters included the number of oocytes retrieved, normally fertilized oocytes, normal fertilization rate, unfertilized oocytes, immature oocytes, degenerated oocytes, and polyspermic fertilization rates. Embryo development parameters included the number of cleaved embryos, cleavage rate, uncleaved oocytes, Day 3 high-quality embryos, blastocysts formed, high-quality blastocysts, blastocysts cultured, and usable embryos. Lastly, embryo transfer and hormone parameters encompassed the number of embryos transferred, estradiol (E2) levels on embryo transfer day, and progesterone levels on embryo transfer day. These data were collected from patient records, laboratory logs, and clinical assessments to support the study’s analysis and model development.

### Key definitions

Infertility: Failure to achieve a successful pregnancy after 12 months or more of regular, unprotected sexual intercourse.

Primary Infertility: A condition in which a woman has never experienced clinical pregnancy despite engaging in regular, unprotected sexual intercourse with her male partner for 12 months or longer.

Secondary Infertility: A condition in which a woman has a history of clinical pregnancy but fails to achieve clinical pregnancy again after 12 consecutive months or more of regular, unprotected sexual intercourse.

Clinical Pregnancy: Defined as a positive urinary hCG and serum β-hCG test 14–16 days after embryo transfer. If hCG levels remain positive and menstruation does not occur, a transvaginal ultrasound performed 56 days after the last menstrual period confirms clinical pregnancy by the presence of a gestational sac in the uterine cavity, containing an embryonic pole with detectable cardiac activity.

### Statistical analysis

Statistical analyses were performed using R software (version 4.2.1) and SPSS software (version 25.0). For continuous variables with a normal distribution, comparisons were conducted using the t-test, and data were expressed as mean ± standard deviation (mean ± SD). For non-normally distributed continuous variables, the Mann-Whitney U test was employed, with data presented as the median and interquartile range [M (P25, P75)]. Categorical variables were analyzed using the χ² test or Fisher’s exact test, and results were expressed as counts (n) and percentages (%). Initially, univariate analysis was performed to assess the statistical significance of each factor influencing pregnancy outcomes. A heatmap was then generated to evaluate correlations among continuous variables and detect potential multicollinearity. All samples were randomly divided into a training set and a validation set at a ratio of 7:3. To address multicollinearity among variables, the LASSO regression model was employed for variable selection. Subsequently, a logistic risk prediction model was developed, and the results were visualized using a nomogram. Model reliability was assessed using the receiver Receiver Operating Characteristic(ROC) curve, calibration curve, Decision Curve Analysis(DCA), and clinical impact curve. A P-value < 0.05 was considered statistically significant.

## Results

### Clinical characteristics

This study included a total of 11,449 infertile patients undergoing IVF/ICSI treatment, with 5,949 patients in the pregnancy group and 5,500 patients in the non-pregnancy group. In this study, a total of 26 variables showed significant differences (P<0.05) between the pregnant and non-pregnant groups (See **Table 1**). These variables include male age, female age, female BMI, duration of infertility, EMT, Day 3 FSH levels, AFC, number of mature MII oocytes, volume, type of fertilization, number of oocytes retrieved, number of normally fertilized oocytes, normal fertilization rate, number of fertilized oocytes or thawed embryos, unfertilized oocytes, Immature oocytes, polyspermic fertilization, number of cleaved embryos, number of Day 3 high-quality embryos, number of blastocysts formed, number of high-quality blastocysts, number of blastocysts cultured, number of usable embryos, number of embryos transferred, E2 levels on embryo transfer day, and progesterone levels on embryo transfer day. These significant differences suggest that these variables are closely associated with clinical pregnancy outcomes. To assess the correlation between continuous variables included in the study, we created a correlation heatmap. The heatmap illustrates the correlations between certain continuous variables, such as between MII oocytes and the number of oocytes retrieved, number of normally fertilized oocytes and “number of fertilized oocytes or thawed embryos”.

**Table 1:** Clinical characteristics analysis of the pregnant and no-pregnant groups.

Characteristics	Overall (n=11449)	No-pregnant (n=5500)	Pregnant (n=5949)	
General Information				
Male age (median [IQR]), y	33.00 [30.00, 37.00]	34.00 [30.00, 38.00]	32.00 [29.00, 36.00]	<0.001
Male BMI (median [IQR]), kg/m²	24.00 [21.72, 26.17]	24.09 [21.80, 26.18]	23.89 [21.63, 26.17]	0.15
Female age (median [IQR]), y	31.80 [28.80, 35.20]	32.80 [29.50, 36.60]	31.00 [28.30, 33.90]	<0.001
Female BMI (median [IQR]), kg/m²	22.03 [20.13, 24.22]	22.21 [20.28, 24.34]	21.83 [20.03, 24.03]	<0.001
Causes of infertility (n, %)				0.539
Male factor	578 (5.0)	292 (5.3)	286 (4.8)	
Female factor	9123 (79.7)	4378 (79.6)	4745 (79.8)	
Couple factor	1120 (9.8)	539 (9.8)	581 (9.8)	
Unexplained	628 (5.5)	291 (5.3)	337 (5.7)	
Type of infertility (n, %)				0.483
Primary infertility	4794(41.9)	2288(41.5)	2510(42.2)	
Secondary infertility	6655 (58.1)	3216 (58.5)	3439 (57.8)	
Duration of infertility (median [IQR]), y	4.00 [2.00, 6.00]	4.00 [2.00, 6.00]	3.00 [2.00, 5.00]	0.002
Female Baseline Clinical Parameters				
EMT (median [IQR]), mm	11.00 [9.00, 13.00]	10.00 [9.00, 12.00]	11.00 [10.00, 13.00]	<0.001
Day3 FSH (median [IQR]), IU/L	4.52 [2.38, 6.26]	4.84 [2.58, 7.29]	3.82 [2.22, 5.03]	<0.001
AFC (median [IQR]), n	10.00 [6.00, 13.00]	8.00 [5.00, 12.00]	10.00 [7.00, 14.00]	<0.001
Number of MII oocyte (median [IQR]), n	9.00 [5.00, 12.00]	7.00 [4.00, 11.00]	10.00 [7.00, 13.00]	<0.001
Male Semen Quality and Functional Parameters				
volume (median [IQR]), ml	2.50 [2.00, 3.50]	2.50 [1.67, 3.50]	2.50 [2.00, 3.50]	0.007
Concentration (median [IQR]), (10^6^/ml)	59.60 [33.30, 101.00]	59.70 [33.00, 101.12]	59.50 [33.50, 101.00]	0.968
Total motility (median [IQR]), %	54.60 [45.00, 64.70]	54.50 [44.80, 64.50]	54.80 [45.10, 65.00]	0.251
Viability (median [IQR]), %	43.80 [34.90, 53.10]	43.60 [34.70, 52.90]	43.95 [35.00, 53.20]	0.127
Abnormal morphology (median [IQR]), %	87.00 [83.00, 90.00]	87.00 [83.00, 90.00]	87.00 [83.00, 90.00]	0.929
Acrosin (median [IQR]), (mIU/10^6^	100.90 [75.80, 146.10]	100.90 [75.50, 146.98]	100.90 [76.10, 145.20]	0.906
DFI (median [IQR]), %	12.00 [9.00, 16.00]	12.00 [9.00, 16.00]	12.00 [9.00, 16.00]	0.749
Type of fertilization (n, %)				0.038
IVF	10123(88.4)	4827(87.8)	5296(89.0)	
ICSI	1326 (11.6)	673 (12.2)	653 (11.0)	
Oocyte Retrieval and Fertilization Parameters				
Number of oocytes retrieved (median [IQR]), n	10.00 [6.00, 14.00]	8.00 [5.00, 12.00]	11.00 [7.00, 14.00]	<0.001
Number of Normally fertilized oocytes (median [IQR]), n	6.00 [3.00, 9.00]	5.00 [3.00, 8.00]	7.00 [5.00, 10.00]	<0.001
Normal fertilization rate (median [IQR]), n	0.73 [0.56, 0.86]	0.70 [0.50, 0.88]	0.75 [0.60, 0.86]	<0.001
Number of fertilized oocytes or thawed embryos (median [IQR]), n	10.00 [6.00, 14.00]	8.00 [5.00, 12.00]	11.00 [7.00, 14.00]	<0.001
Unfertilized oocytes (median [IQR]), n	1.00 [0.00, 2.00]	1.00 [0.00, 2.00]	1.00 [0.00, 2.00]	0.002
Immature oocytes (median [IQR]), n	0.00 [0.00, 1.00]	0.00 [0.00, 1.00]	1.00 [0.00, 2.00]	<0.001
Degenerated oocytes (median [IQR]), n	0.00 [0.00, 0.00]	0.00 [0.00, 0.00]	0.00 [0.00, 0.00]	0.88
Polyspermic fertilization (median [IQR]), n	0.00 [0.00, 1.00]	0.00 [0.00, 1.00]	0.00 [0.00, 1.00]	<0.001
Embryo Development Parameters				
Number of cleaved embryos (median [IQR]), n	9.00 [6.00, 13.00]	8.00 [4.00, 12.00]	11.00 [7.00, 14.00]	<0.001
Cleavage rate (median [IQR]), %	1.00 [1.00, 1.00]	1.00 [1.00, 1.00]	1.00 [1.00, 1.00]	0.25
Number of uncleaved oocytes (median [IQR]), n	0.00 [0.00, 0.00]	0.00 [0.00, 0.00]	0.00 [0.00, 0.00]	0.053
Number of Day 3 high-quality embryos (median [IQR]), n	1.00 [1.00, 3.00]	1.00 [0.00, 2.00]	2.00 [1.00, 4.00]	<0.001
Number of blastocysts formed (median [IQR]), n	4.00 [2.00, 5.00]	3.00 [1.00, 5.00]	4.00 [3.00, 6.00]	<0.001
Number of high-quality blastocysts (median [IQR]), n	3.00 [1.00, 5.00]	2.00 [1.00, 4.00]	3.00 [2.00, 5.00]	<0.001
Number of blastocysts cultured (median [IQR]), n	0.00 [0.00, 6.00]	0.00 [0.00, 3.00]	0.00 [0.00, 6.00]	<0.001
Number of usable embryos (median [IQR]), n	3.00 [2.00, 5.00]	2.00 [2.00, 4.00]	4.00 [3.00, 6.00]	<0.001
Embryo Transfer and Implantation Parameters				
Number of embryos transferred (median [IQR]), n	2.00 [2.00, 2.00]	2.00 [1.00, 2.00]	2.00 [2.00, 2.00]	<0.001
E2 on ET day (median [IQR]), pmol/L	919.00 [605.00, 1260.00]	842.10 [545.00, 1165.00]	991.00 [662.00, 1328.03]	<0.001
Progesterone on ET day (median [IQR]), nmol/L	40.00 [40.00, 40.00]	40.00 [40.00, 40.00]	40.00 [40.00, 40.00]	<0.001

IQR, interquartile range; BMI, body mass index; EMT, endometrial thickness; FSH, follicle-stimulating hormone; AFC, antral follicle count; MII, metaphase II; DFI, DNA fragmentation index; IVF, in vitro fertilization; ICSI, intracytoplasmic sperm injection; E2, estradiol; ET day, embryo transfer day.

### Variable selection

**Figure 2:**
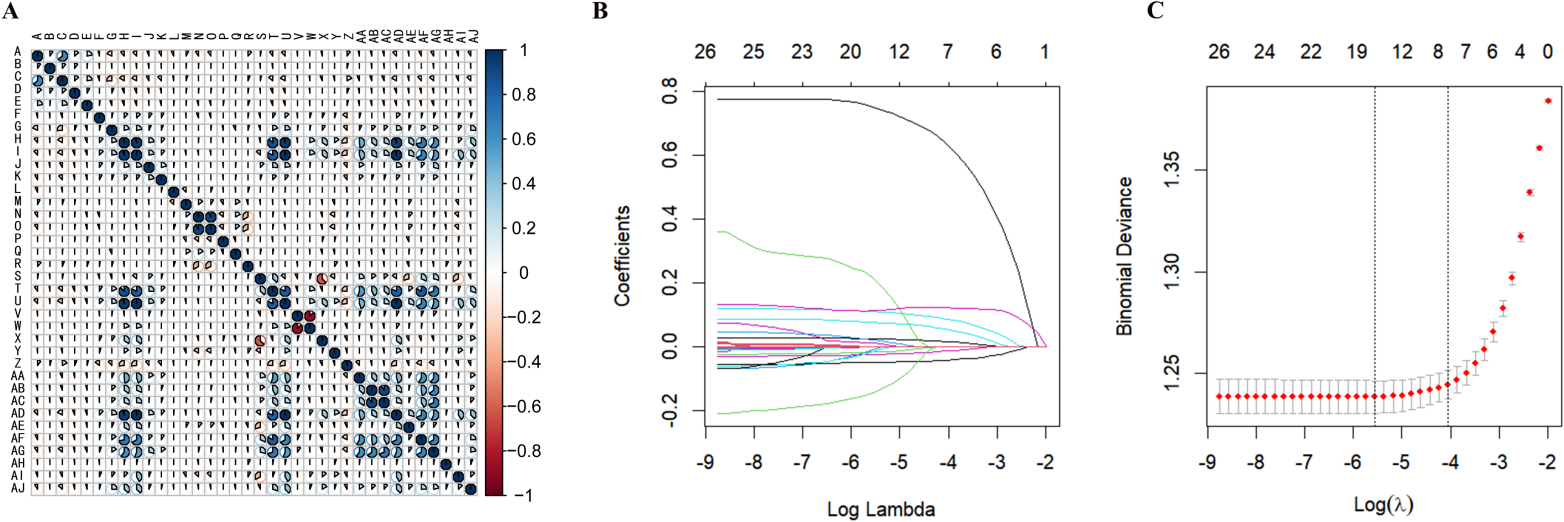
Feature selection and LASSO regression analysis. **(A)** Correlation Heatmap: Displays the correlation matrix among continuous variables. The color gradient indicates varying degrees of correlation, with blue representing positive correlations and red indicating negative correlations. **(B)** LASSO Coefficient Profiles of the 26 Variables: The coefficient profile plot is presented against the log(λ) sequence. The optimal penalty coefficient (λ) was determined through tenfold cross-validation using the minimization criterion. **(C)** The binomial deviance curve was plotted against log(λ), with dotted vertical lines drawn based on the 1 standard error criterion. Eight variables with nonzero coefficients were identified using the 1 standard error criterion.

The heatmap demonstrates correlations among certain continuous variables, indicating the potential presence of multicollinearity among the selected variables (**Figure 2A**). LASSO regression was applied to screen the 26 candidate variables, ultimately identifying 8 key predictive variables (**Figures 2B, C**): female age, Day 3 FSH levels, AFC, EMT, number of high-quality blastocysts, number of available embryos, number of transferred embryos, and male age. These variables exhibited significant associations with clinical pregnancy outcomes and were incorporated into the final predictive model.

### Nomogram construction

**Figure 3:**
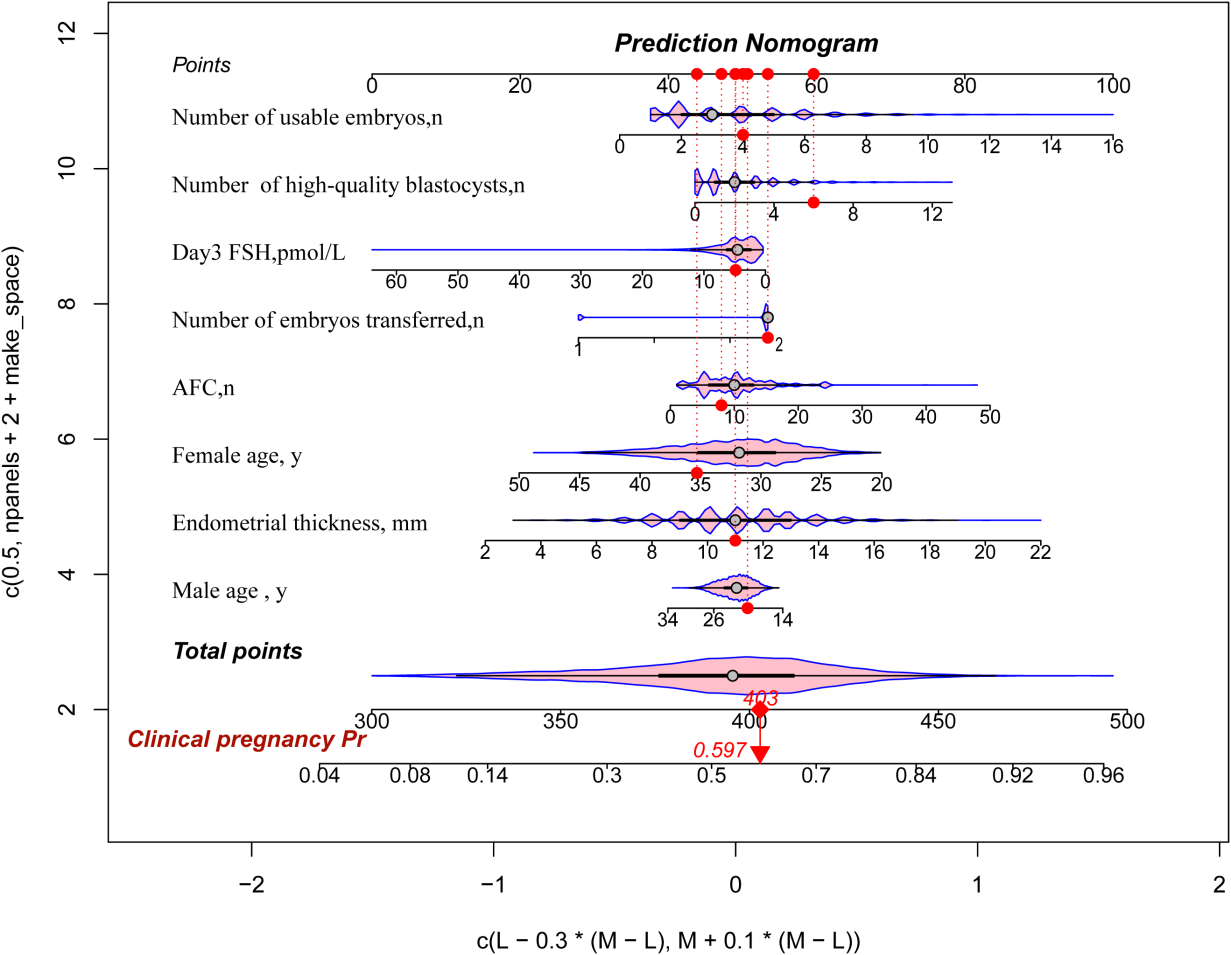
Nomogram for predicting clinical pregnancy for infertility treatment with IVF/ICS. This figure presents a predictive model constructed based on multivariate analysis, with a nomogram used to assess the probability of clinical pregnancy. The top axis (Points) indicates the score assigned to each variable. Predictive variables include the number of available embryos, number of high-quality blastocysts, Day 3 FSH level, number of transferred embryos, antral follicle count (AFC), female age, endometrial thickness, and male age. The specific values for each variable are displayed along the horizontal axis. Total Points: The scores for all variables are summed, and the total is located on the bottom axis of the figure.

Based on multivariable logistic regression analysis, we constructed a nomogram to predict the clinical pregnancy probability in IVF/ICSI patients. The nomogram includes 8 key indicators, and by assigning a score to each variable and calculating the total score, it provides a visual representation of the pregnancy probability for individual patients, serving as an effective decision-making tool for clinicians. For instance, a female aged 35 years (44 points), a male aged 20 years (51 points), Day 3 FSH levels of 5 µmol/L (47 points), an AFC of 8 (48 points), an EMT of 11 mm (49 points), 6 high-quality blastocysts (61 points), 4 available embryos (50 points), and 2 transferred embryos (53 points) would yield a total score of 403, predicting a probability of 0.597 (**Figure 3**).

### Nomogram accuracy evaluation and validation

**Figure 4:**
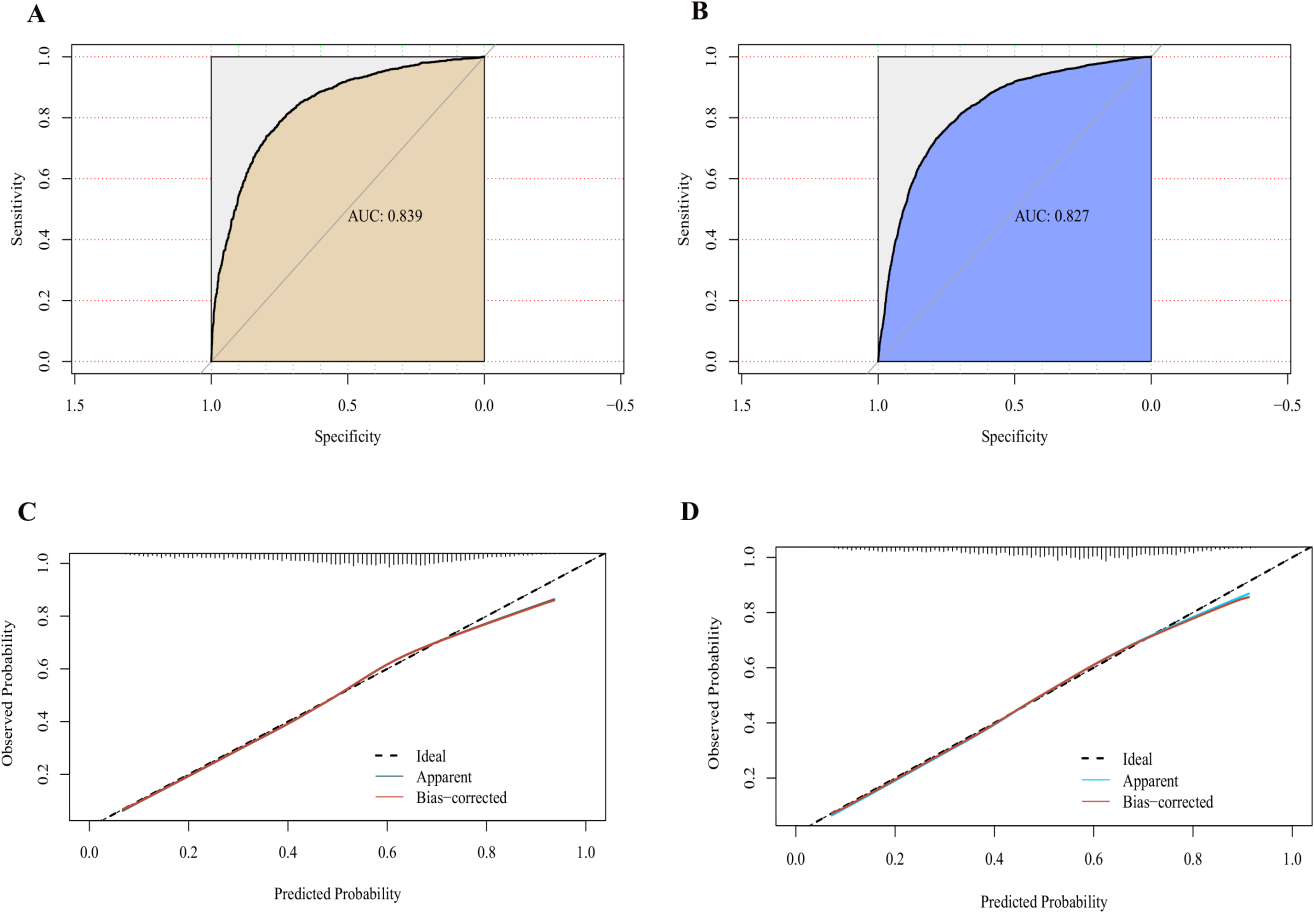
Performance evaluation of the clinical pregnancy outcome prediction model for infertility treatment with IVF/ICSI. **(A)** ROC Curve for the Training Set: Demonstrates the discriminative performance of the prediction model on the training set, with an AUC of 0.839. The X-axis represents specificity, and the Y-axis represents sensitivity. **(B)** ROC Curve for the Validation Set: Illustrates the model’s discriminative performance on the validation set, with an AUC of 0.827. The X-axis represents specificity, and the Y-axis represents sensitivity. **(C)** Calibration Curve for the Training Set: Shows the agreement between the predicted probabilities and observed probabilities in the training set. The ideal curve is depicted as a dashed line, while the model’s actual performance is represented by the solid line. **(D)** Calibration Curve for the Validation Set: Displays the agreement between the predicted probabilities and observed probabilities in the validation set.

The model’s discriminatory ability was assessed using the ROC curve, with AUC values of 0.839 [95% CI (0.825, 0.852)] for the training set (**Figure 4A**) and 0.827 [95% CI (0.817, 0.835)] for the validation set (**Figure 4B**), indicating excellent discrimination. The calibration curve showed that the predicted probabilities were highly consistent with the actual pregnancy rates (**Figures 4C, D**), and the Hosmer-Lemeshow test yielded P-values of 0.184 and 0.723, respectively, suggesting good calibration and reliable predictions.

### Clinical utility assessment

**Figure 5:**
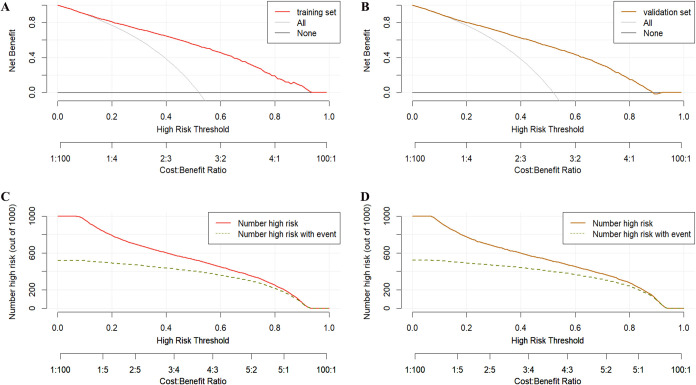
Decision curve analysis and clinical impact curves for the clinical pregnancy outcome prediction model in IVF/ICSI. **(A)** Decision Curve for the Training Set: Demonstrates the net benefit of the model across varying high-risk thresholds in the training set. The red line represents the net benefit of the model, while “All” and “None” serve as baselines, assuming all or no patients are considered high risk, respectively. **(B)** Decision Curve for the Validation Set: Illustrates the net benefit of the model across different thresholds in the validation set. **(C)** Clinical Impact Curve for the Training Set: Shows the number of patients classified as high risk and the corresponding number of true positives at different probability thresholds in the training set. **(D)** Clinical Impact Curve for the Validation Set: Displays the number of patients classified as high risk and the true positives at varying thresholds in the validation set.

DCA demonstrated that the model exhibited high net benefits across different probability thresholds, outperforming the “full prediction of pregnancy” or “full prediction of non-pregnancy” baseline models in both the validation and test sets (**Figures 5A, B**). Additionally, the clinical impact curve highlighted the model’s ability to accurately identify high-risk patients and effectively differentiate actual positive cases at varying thresholds, emphasizing its substantial clinical utility (**Figures 5C, D**).

## Discussion

This study retrospectively analyzed clinical data from a single center in Yunnan Province involving patients undergoing IVF/ICSI treatment for infertility. The research aimed to identify risk factors influencing pregnancy outcomes and developed a predictive model based on male age, AFC, Day 3 FSH level, endometrial thickness, female age, number of usable embryos, number of high-quality blastocysts, and number of embryos transferred. Validation using ROC curve, calibration curve, DCA curve, and clinical impact curve demonstrated the model’s high accuracy and clinical utility. It is hoped that this model can provide evidence-based guidance for the clinical management and treatment of patients undergoing IVF/ICSI for infertility.

Female age is a key factor affecting the outcomes of IVF/ICSI treatment. Advanced maternal age poses great challenges in reproductive medicine, primarily manifested as a gradual decline in both the quantity and quality of oocytes, accompanied by a decrease in overall fertility potential (20, 21). As women age, ovarian reserve significantly decreases, and the remaining oocytes become more sensitive to oxidative stress and mitochondrial dysfunction (22–24). This age-related decline in oocyte quantity and quality leads to reduced fertilization rates, impaired embryo development, and poor pregnancy outcomes in ART, especially IVF. However, male partners also contribute half of the genetic material for embryos, so sperm quality plays an equally important role in embryo quality and affects ART outcomes (25). According to WHO standards, optimal semen quality values occur between the ages of 30 and 35, with the most significant decline occurring after the age of 55 (26, 27). Abnormalities in various indicators of semen parameters may reduce fertility (28, 29). Increasing evidence suggests that advanced paternal age is associated with decreased fertility and an increased risk of natural miscarriage (30). Additionally, older fathers can lead to male infertility and poor ART outcomes (31, 32). Interestingly, studies showed that oocytes from younger women can partially reverse the decline in sperm quality due to increasing male age, thereby improving LBRs. As oocytes age, their repair mechanisms weaken, and the decline in sperm quality adversely affects fertility outcomes (33). Therefore, understanding the ages of both partners and the interaction between their ages is crucial for optimizing IVF/ICSI treatment plans and patient counseling.

EMT is a frequently monitored indicator during the IVF/ICSI process and is also an indirect indicator of endometrial receptivity. A normally shaped endometrium is one of the essential requirements for the success of ART. Current available evidence suggests that 50% to 70% of implantation failures are related to poor endometrial receptivity, with an ideal endometrial thickness of 7 mm or thicker for embryo transfer (34, 35). A thin endometrium in assisted reproduction is typically defined as an endometrial thickness <7mm or <8mm (35). A thin endometrium often leads to low implantation rates, reproductive disorders, and termination of ART plans. Additionally, there is an increased risk of early miscarriage, preterm birth, and low birth weight children (36). This may be because a thin endometrium brings the embryo closer to the spiral arteries and exposes it to higher oxygen concentrations, which is known to be harmful to embryonic development. However, the relationship between increased EMT (>14 mm) and pregnancy outcomes remains controversial. Weissman et al. demonstrated that women with thickened endometrium had lower implantation rates and pregnancy rates, with increased miscarriage rates (37). In contrast, Zhang et al. indicated that increased EMT often improves the clinical pregnancy rate (CPR) of IVF (37). Therefore, there is still a lack of consensus on the impact of endometrial thickening on IVF/ICSI pregnancy outcomes.

FSH is an important reproductive hormone that plays a key role in women’s fertility. In particular, the baseline serum FSH level on day 3 is considered an important indicator reflecting the ovarian reserve function status in women, which also significantly impacts the outcomes of ART treatment (38). A study reported that among 8019 IVF cycles using autologous oocytes, women with baseline FSH levels exceeding 18 mIU/mL failed to achieve live birth (39). Another early study reported that among 1750 women with baseline FSH levels below 20 mIU/mL, the pregnancy rate was 16.5%, whereas for those with a single baseline FSH ≥20 mIU/mL during the treatment cycle, the pregnancy rate was only 5.6% (40). A research conducted at a large IVF center in the UK showed that the LBR for women with FSH levels exceeding 20 mIU/mL was only 3.0% (41). This may be due to high FSH levels reflecting a decline in ovarian function, leading to reduced responsiveness to ovulation induction medications, thereby affecting treatment outcomes. Therefore, clinicians should fully consider the patient’s day 3 FSH level indicator when formulating ART treatment plans.

AFC reflects another aspect of ovarian reserve, and it assesses the number of available follicles in the ovaries through ultrasound examination. AFC has good predictive value for several important IVF outcomes, with studies showing a linear relationship between AFC and the number of retrieved oocytes (42), as well as its correlation with the ovarian response to gonadotropins (43). However, reports on the correlation with CPRs and LBRs vary widely (44–47). It has also been suggested that determining AFC may help in identifying appropriate stimulation protocols (45). Therefore, AFC, as a simple, non-invasive, and intuitive assessment tool, is widely used to predict women’s responses and pregnancy outcomes in IVF treatment.

The number of available embryos and the number of high-quality blastocysts are critical indicators that reflect the embryonic development during IVF/ICSI treatment. The number of available embryos reflects the development of fertilized eggs and is the basis for determining the number of embryos to be transferred. Generally speaking, the more available embryos there are, the better the quality of the fertilized eggs and the stronger the developmental potential, thereby increasing the chances of implantation and pregnancy after transfer (48). The number of high-quality blastocysts more directly reflects the quality of the embryos. Research has found that transferring high-quality blastocysts can significantly improve CPRs and LBRs. High-quality blastocysts have better developmental potential and implantation ability, and transferring such embryos can increase the success rate of treatment (49). In addition, the number of transferred embryos is also a key indicator, as it directly determines the chances of implantation. The pregnancy rate and LBR of transferring two embryos are significantly higher than that of transferring a single embryo (50). However, it is also important to note that transferring too many embryos may increase the risk of multiple pregnancies, so a balance needs to be struck based on specific circumstances. In summary, these embryo-related indicators during treatment reflect the quality of eggs and sperm as well as the level of laboratory operations, thus having a significant impact on the final treatment outcomes. Clinicians should make full use of these indicators to optimize treatment plans and improve the success rate of treatment.

This study has several innovative aspects. First, it specifically focuses on the clinical pregnancy outcomes of fresh embryo transfer, rather than a mixed analysis of fresh and frozen-thawed embryos. This approach minimizes the potential interference caused by embryo freezing and thawing processes on pregnancy outcomes, making the findings more directly applicable to treatment strategies for fresh embryos. Second, we systematically and comprehensively analyze clinical characteristics and laboratory indicators associated with pregnancy outcomes during IVF/ICSI treatment, ensuring the inclusion of a wide range of variables to enhance scientific rigor. Lastly, this study is one of the few large-scale predictive model studies in the field of IVF/ICSI, involving a substantial sample size of 154,307 cases. This not only improves the predictive power of the model but also provides greater statistical reliability to the findings.

However, the study is not without limitations. First, as a single-center retrospective study, it may be subject to selection bias related to the specific region, ethnicity, or socio-economic and cultural context of the study population. Although the sample size is large, the data’s single-source nature may result in limited heterogeneity, making the model less adaptable to high-dimensional interactions or varying variable distributions in other centers. Therefore, future prospective studies with larger, more diverse samples are needed to validate the accuracy and generalizability of the conclusions drawn from this research.

## Conclusion

This study highlighted key factors influencing clinical pregnancy outcomes in IVF/ICSI treatment, including female age, male age, AFC, Day 3 FSH levels, endometrial thickness, the number of usable embryos, the number of high-quality blastocysts, and the number of embryos transferred. The developed model provides a scientifically robust decision-support tool to assist clinicians in optimizing infertility treatment through IVF/ICSI.

## Data Availability

The raw data supporting the conclusions of this article will be made available by the authors, without undue reservation.
